# Parent–adolescent communication on sexual and reproductive health and the utilization of adolescent-friendly health services in Kailali, Nepal

**DOI:** 10.1371/journal.pone.0246917

**Published:** 2021-02-19

**Authors:** Bharat Raj Bhatta, Junko Kiriya, Akira Shibanuma, Masamine Jimba

**Affiliations:** Department of Community and Global Health, Graduate School of Medicine, The University of Tokyo, Bunkyō, Japan; National University of Singapore, SINGAPORE

## Abstract

**Background:**

Adolescents are vulnerable to various sexual and reproductive health (SRH) problems such as unintended pregnancy, HIV or other sexually transmitted infections (STIs), and unsafe abortion. Adolescent-friendly health services offer SRH services such as SRH counseling, contraceptive services, STI and HIV services, and abortion-related services, which may help prevent these risks. Parent–adolescent communication about SRH prevents adolescents from adopting unhealthy SRH practices. However, its association with the utilization of SRH services is less known. Therefore, this study examined the association between parent–adolescent communication on SRH issues and the utilization of adolescent-friendly health services in Nepal.

**Methods:**

This was a school-based, cross-sectional study conducted in Kailali district, Nepal, among students aged 15–19 years in Grade 11 and 12 from seven schools. We used multivariable logistic regression analysis to examine the association between parent–adolescent communication and service utilization.

**Results:**

We analyzed the data from 594 students. Students with a higher score of parent–adolescent communication on SRH were significantly more likely to use adolescent-friendly health services (adjusted odds ratio, AOR: 1.70, 95% Confidence Interval, CI: 1.29–2.23, p<0.001). Those who reported having engaged in sexual intercourse in the past year were more likely to use services than those who did not (AOR: 29.11, 95% CI: 13.65–62.08, p<0.001). Those who belonged to the Janajati ethnic group were more likely to use these services than those from the Brahmin/Chhetri ethnic group (AOR: 2.86, 95% CI: 1.28–6.42, p = 0.01). Those living alone were less likely to use services than those living with both parents (AOR: 0.12, 95% CI: 0.02–0.66, p = 0.01).

**Conclusion:**

Students with a higher score on parent–adolescent communication on SRH were more likely to use adolescent-friendly health services. Thus, parental involvement in SRH communication could contribute to the use of adolescent-friendly health services and ultimately prevent negative SRH outcomes among students in late adolescence.

## Introduction

As compared to any other age group, adolescents are more vulnerable to various sexual and reproductive health (SRH) problems [1]. Globally, they constitute a population of 1.2 billion, and more than 80% of them live in low- and middle-income countries [1, 2]. They are particularly vulnerable to SRH problems such as unintended pregnancy, HIV or other sexually transmitted infections (STIs), and unsafe abortion. STIs affect two million adolescents globally, and they are the second leading cause of death among this age group [3]. Similarly, complications from adolescent pregnancy and childbirth are the leading cause of death among adolescent girls aged 15–19 years [1]. Thus, to prevent these risks, it is vital to ensure that this population has access to SRH services [4].

Risky sexual behaviors and reproductive health problems during adolescence have long-term impacts that continue to affect them throughout their life [5]. The Use of SRH services can prevent and reduce these risks [4, 6]. As SRH is a sensitive issue in several countries, adolescent-friendly health services could encourage adolescents to share their problems and receive appropriate solutions [7, 8].

Adolescent-friendly health services, which are provided with adolescents’ informed consent, offer comprehensive SRH services while ensuring confidentiality, privacy, and respect [7, 9]. In 1994, the International Conference on Population and Development identified adolescent-friendly health services as a key strategy for meeting adolescents’ health needs, especially those related to SRH. These services are affordable, accessible, appropriate, and acceptable, and they are provided by trained health professionals [7]. They include SRH counseling; contraceptive counseling; STI and HIV counseling, testing, treatment and care; prenatal and postnatal care; sexual abuse counseling; and abortion-related services [7, 10].

Despite the availability of such services, adolescents continue to face several unmet needs related to SRH, such as those related to the use of contraceptives, and they experience barriers to accessing these services [4, 11]. These obstacles mainly exist because of their inadequate knowledge and experience about the services required and the lack of confidentiality [12]. For SRH services, they face additional barriers such as shyness, ignorance, quality of care, cost, inconvenient time, location of youth-related services, and judgmental behavior of healthcare providers [13, 14].

Parents are one of the key protective factors for adolescents’ health. They greatly influence their children’s overall attitudes and behaviors towards health, including SRH. They can potentially be an important source of SRH information to their children [15, 16]. Parent–adolescent communication about SRH prevents adolescents from adopting unhealthy SRH practices and promotes their health [17, 18]. Furthermore, such communication is associated with later sex initiation [19] and greater ability to negotiate condom use [20].

Adolescents’ utilization of SRH services is associated with several factors. The most common ones include age, knowledge about reproductive health, cost, distance to the facility, and knowledge about the available services [21, 22]. In addition, parent–adolescent communication was associated with increased voluntary counseling and testing (VCT) service utilization among sexually active adolescents in Ethiopia [23]. However, the association of parent–adolescent communication with the utilization of adolescent-friendly health services is less known globally.

Adolescents constitute nearly a quarter of the 28 million population of Nepal [24]. The mean age at the first sexual encounter among youths and adolescents was 17.5 years in 2011 [25]. Only 58.6% of adolescent students aged 15–19 years used a condom during their first sexual intercourse [26]. Further, risky sexual behaviors including having more than one sexual partner, engaging in sexual intercourse with a sex worker, and inconsistent condom use, were prevalent in this population [26, 27].

In Nepal, children usually live with their parents. However, it is uncommon and uncomfortable for family members to discuss issues related to SRH. Primarily due to the lack of adequate communication, followed by the lack of proper information, education, and counseling, adolescents remain confused at this age [14, 28]. They may tend to depend on their close friends, who may not always be a reliable source of information and support [28]. Such situations expose adolescents to various health risks, and therefore, it is crucial to provide them appropriate support and services

Nepal initiated a national adolescent SRH program in 2008, which aimed to improve adolescents’ SRH by focusing on providing adolescent-friendly health services through existing government health facilities. In 2016, 1,134 health facilities delivered such services in 63 districts of Nepal [29]. These facilities mainly focus on providing SRH services such as family planning; STI and HIV services; safe abortion; SRH counseling; management of sexual- and gender-based violence; and information, education, and services for general health concerns. Adolescents can access these services by visiting these facilities. These services are delivered by trained staff in a confidential and non-judgmental manner. The facilities also provide educational materials to promote adolescent SRH [29–31]. Despite the provision of these resources, adolescents in Nepal lack access to proper information and services on SRH, and their service utilization ranges merely from 9.2% [32] to 24.7% [33]. Further, service utilization varies by the location, availability, and accessibility of related services [33].

Limited evidence is available regarding the association between parent–adolescent communication and the utilization of SRH services. Therefore, this study was conducted to examine the association between parent–adolescent communication on SRH issues and the utilization of adolescent-friendly health services among adolescent students in Nepal.

## Materials and methods

### Study design and settings

We conducted a school-based, cross-sectional study in Kailali district of Nepal, using a self-administered questionnaire. Located in Sudurpaschim province, Kailali is one of the districts that implements an adolescent SRH program through adolescent-friendly health centers. The district had a total population of 775,709 individuals, with an adolescent population of 202,152 in 2011 [34, 35]. The literacy rate in the district was 66.3% in 2015. The gross secondary school enrolment rate for Grades 11–12 in the district was 55% in 2017 [34, 36]. Adolescent students from other districts of the Sudurpaschim province also receive secondary and higher education in schools in Kailali [37]. Therefore, students from the schools in this district also represent students in late adolescence in the province.

### Sample size and sampling procedure

We calculated the required sample size using OpenEpi version 3.0 based on a previous study conducted in Ethiopia, with an odds ratio value of 4.9 [23]. We set the power of the study at 80%, level of significance at 5%, and confidence interval at 95%, and then calculated the minimum sample size as 121, with a 10% non-response rate. We assumed within-school-intra-class correlation coefficient of 0.1 for health outcomes [38]. We considered each class as a cluster, and the average number of students in a class was 40. Then we calculated the design effect as 4.9, using the formula Deff = 1+(m-1)×p, where m represents the number of students in each class, and p is the intra-class correlation coefficient [38]. After multiplying the minimum sample size by the design effect, the required number of students was 593. However, to account for missing data owing to the sensitive nature of the topic, we estimated the sample size as 640.

We obtained the list of schools from the District Education Office in Kailali district. The district had 18 adolescent-friendly health centers, and these locations had 51 secondary schools, out of which we randomly selected seven schools using computer-generated random numbers. To recruit students from each school, we randomly chose one class from each grade in a school using the lottery method. We then approached all the students in the selected class for participation in the study. We recruited students based on the following inclusion criteria: being students in late adolescence, aged 15–19 years, studying in Grade 11 and 12 of secondary school, and being present on the day of data collection. Adolescence is defined as the age of 10 to 19 years [4, 39].

### Outcome variable

The main outcome of this study was the utilization of adolescent-friendly health services. The definition of the utilization of the adolescent-friendly health services was the use of any one of the following SRH services from adolescent-friendly health centers within the past one year [7, 40, 41]:

- Family planning or contraceptive-related counseling and services
- SRH information and counseling such as pubertal changes and sexual relationships
- STI/HIV counseling, testing, or treatment services
- Pregnancy-related services (pregnancy test/antenatal care examination or counseling)
- Abortion-related services
- Delivery or postpartum care related counseling or services

We measured service utilization as a dichotomous variable (yes or no) and further asked students about the types of service used during their last visit. We used a structured questionnaire adapted from a previous study conducted in Nepal [32], and the WHO-recommended questionnaire for adolescents [42].

### Exposure variable

The exposure variable of this study was parent–adolescent communication on SRH. We asked adolescents whether they and their parents had discussed the following seven SRH issues within the past year: 1) pubertal changes, 2) menstruation, 3) safe sex, 4) unintended pregnancy, 5) family planning/contraception, 6) STI/HIV, and 7) condom use [43, 44]. The response was either yes or no. We counted the number of “yes” responses as the total score. The total score ranged from zero to seven, with higher scores indicating communication on more topics. We used the questionnaire based on a previous study conducted in Ethiopia on parent–adolescent communication [43]. Two bilingual researchers translated the English version of the questionnaire to the Nepali language, followed by back translation into English to ensure quality and consistency.

### Covariates

We selected the covariates based on previous studies related to SRH service utilization [21, 23, 32].

### Sexual behaviors

Questions pertaining to sexual behaviors explored whether the students had ever engaged in sexual intercourse, and whether they had engaged in sexual intercourse in the past 12 months. We adopted questions from a global school-based student health survey available in Nepali language [45]. We included this variable because adolescents who had engaged in sexual intercourse recently were found to be more likely to use the SRH services [32].

### Sociodemographic characteristics

Sociodemographic variables included sex, age, ethnicity, religion, marital status, mother’s education level, father’s education level, parents’ economic status (wealth quintile measured using the wealth index from data on the household’s ownership of selected assets), and living arrangement (living with parents, others, or alone). We adopted questions from the Nepal Adolescents and Youth Survey, and Nepal Demographic and Health Survey [25, 46]. We included sex, age, and parents’ education as they could be associated with service utilization [23, 32]. Sociocultural factors such as ethnicity and religion may also influence the knowledge about and use of services. We included the economic status as it affects access to information and knowledge of SRH services [21], which can in turn affect service utilization. Similarly, living arrangement may affect the knowledge and utilization of services [21, 23].

### Data collection

We pretested the questionnaire among 40 students in a school different from the sampled schools in the same locality. Based on the pretest results, we modified the wording of some questions and made the instructions for filling the questionnaire clearer. We hired and trained three research assistants with an academic background in public health or nursing. The main researcher (BRB) and research assistants collected data in September, 2017. We visited schools and requested the students to complete the self-administered questionnaire in the halls or classrooms, in front of one of the research assistants or the main researcher. Students took about 35 minutes to complete the questionnaire. The students returned the completed questionnaire in an envelope provided, to ensure confidentiality and anonymity.

### Data analysis

Research assistants entered the data in EpiData version 3.1 and we exported the data to Stata version 13.1 for analyses. We used the Chi-square test, Fisher’s exact test, independent sample t-test and one-way analysis of variance (ANOVA) to describe and understand parent–adolescent communication and service utilization. In the bivariate analysis of two categorical variables, we used the Chi-square test if the numbers of students in all the combinations of categories were five or more, and otherwise, we used Fisher’s exact test. In the bivariate analysis of a categorical variable and a continuous variable, we used an independent sample t-test to compare the means of a continuous variable by two categories in a categorical variable and one-way ANOVA for more than two categories. We used multivariable logistic regression analysis to examine the association between parent–adolescent communication and service utilization adjusted for covariates. We also used multilevel logistic regression analysis with the random intercept at the classroom level to see any variation across classrooms. We checked multicollinearity in the model, and any variable with a variance inflation factor (VIF) value above 10 was excluded from the analyses. Statistical significance was set at 0.05.

### Ethics

We obtained an ethics approval from the Research Ethics Committee, Graduate School of Medicine, The University of Tokyo (No.11656) and the Nepal Health Research Council (No. 271/2017). District Education Office and the selected schools also provided written permission. After receiving their approval, research assistants and the main researcher distributed an information sheet and informed consent sheet for parents/guardians through the students, in coordination with the school teachers. We requested the students to submit their parental/guardians’ consent before starting data collection, and they brought the consent sheets back on the data collection day. Participation in this study was voluntary, and we obtained written assent from the students.

## Results

Of the 662 students approached, 21 did not return the parental or guardian’s consent, and we excluded them. We also excluded 7 students because they were older than 19 years, and 2 as they refused to participate. After excluding these 30 students, 632 students were available for the study, of which we further excluded 38 due to incomplete or missing data. Thus, we analyzed data from 594 questionnaires.

### General characteristics of students

Table 1 shows the sociodemographic characteristics of the students (n = 594). Their mean age was 17.4 (SD 1.0) years and 58% of them were female. Seventy-two percent of them were from public schools and nearly 100% of them were unmarried. Further, 52%, 38.6%, and 9.3% belonged to the Brahmin/Chhetri, Janajati, and Dalit ethnic groups, respectively. Seventy percent of them lived with both parents, while 13.1%, 9.9%, and 6.6% lived with a single parent (either father or mother only), alone, and with others (siblings, relatives, or friends), respectively.

**Table 1:** General characteristics of students (n = 594).

Variables		n	%
**Age** (in years) range 15–19 years; Mean (SD)		17.4 (1.0)	
**Sex**	Male	252	42.4
	Female	342	57.6
**Grade**	11	311	52.4
	12	283	47.6
**Type of school**	Public	426	71.7
	Private	168	28.3
**Marital status**	Unmarried	585	98.5
	Married	9	1.5
**Residence**	Rural	91	15.3
	Urban	503	84.7
**Religion**	Hindu	561	94.4
	Christian/Muslim/Buddhist	33	5.6
**Ethnicity**	Brahmin/Chhetri	310	52.1
	Janajati	229	38.6
	Dalit	55	9.3
**Father’s education** (n = 590)	No education	67	11.4
	Grade 1 to 10	403	68.3
	Grade 11 and above	120	20.3
**Mother’s education** (n = 592)	No education	153	25.8
	Grade 1 to 10	394	66.6
	Grade 11 and above	45	7.6
**Wealth quintile**	Lowest	119	20.0
	Second lowest	120	20.2
	Middle	118	19.9
	Second highest	120	20.2
	Highest	117	19.7
**Living arrangement**	With both parents	418	70.4
	With single parent (either father or mother only)	78	13.1
	Alone	59	9.9
	Others (with siblings, other relatives or friends)	39	6.6

SD: Standard Deviation

### Parent–adolescent communication on SRH

Table 2 shows the mean parent–adolescent communication scores by general characteristics (unadjusted analysis). Female students had a higher mean communication score than did male students. Those who belonged to the Janajati ethnic group had a higher communication score than those from the Brahmin/Chhetri and Dalit ethnic groups. There were no significant differences in the mean communication score based on religion, parents’ education, living arrangement, and wealth quintile.

**Table 2:** Parent–adolescent communication scores by general characteristics (n = 594).

Variables	Mean	SD	Test statistic	p-value	df
Sex*			-3.89	**<0.001**	592
Male	0.6	1.0			
Female	0.9	1.2			
Grade*			2.45	**0.015**	592
11	0.9	1.2			
12	0.7	1.0			
Type of school*			4.24	**<0.001**	592
Public	0.9	1.2			
Private	0.5	0.9			
Marital status*			2.38	**0.018**	592
Unmarried	0.8	1.1			
Married	1.7	1.0			
Residence*			2.13	**0.033**	592
Rural	1.0	1.1			
Urban	0.7	1.1			
Religion*			0.11	0.909	592
Hindu	0.8	1.1			
Christian/Muslim/Buddhist	0.8	1.3			
Ethnicity**			10.72	**<0.001**	2
Brahmin/Chhetri	0.6	1.0			
Janajati	1.0	1.3			
Dalit	0.7	1.0			
Father’s education** (n = 590)			0.10	0.905	2
No education	0.8	1.3			
Grade 1 to 10	0.8	1.1			
Grade 11 and above	0.8	1.2			
Mother’s education** (n = 592)			0.72	0.486	2
No education	0.7	1.1			
Grade 1 to 10	0.8	1.2			
Grade 11 and above	0.6	0.9			
Living arrangement**			2.26	0.081	3
With both parents	0.8	1.2			
With single parent	0.7	1.1			
Alone	0.5	0.9			
With others (with siblings, other relatives or friends)	0.6	1.0			
Wealth quintile**			1.44	0.219	4
Lowest	0.9	1.2			
Second lowest	0.9	1.2			
Middle	0.8	1.2			
Second highest	0.7	1.1			
Highest	0.6	1.0			

SD: Standard Deviation; df: Degree of freedom

* independent samples t-test

** one-way analysis of variance (ANOVA)

Table 3 presents the findings on the topics of parent–adolescent communication on SRH, as reported by the students. Evidently, 17.8%, 16.2%, and 14.8% reported that they discussed about menstruation, pubertal changes, and STI/HIV with their parents, respectively. Further, the topics on menstruation and unintended pregnancy were discussed more often by female students than male students. There were no significant gender differences on other communication topics.

**Table 3:** Parent–adolescent communication topics on SRH (n = 594).

Communication topics	N (%)*	Male		Female		Chi-square statistic	p-value^ǂ^
		n	%	N	%		
**Pubertal changes**						3.04	0.081
Yes	96 (16.2)	33	13.1	63	18.4		
No	498 (83.8)	219	86.9	279	81.6		
**Menstruation**						51.10	**<0.001**
Yes	106 (17.8)	12	4.8	94	27.5		
No	488 (82.2)	240	95.2	248	72.5		
**Safe sex**						0.16	0.685
Yes	35 (5.9)	16	6.3	19	5.6		
No	559 (94.1)	236	93.7	323	94.4		
**Unintended pregnancy**						4.97	**0.026**
Yes	59 (9.9)	17	6.7	42	12.3		
No	535 (90.1)	235	93.3	300	87.7		
**Contraceptives/Family planning**						0.91	0.341
Yes	60 (10.1)	22	8.7	38	11.1		
No	534 (89.9)	230	91.3	304	88.9		
**STI/HIV**						1.55	0.213
Yes	88 (14.8)	32	12.7	56	16.4		
No	506 (85.2)	220	87.3	286	83.6		
**Condom**						3.45	0.063
Yes	19 (3.2)	12	4.8	7	2.0		
No	575 (96.8)	240	95.2	335	98.0		

* N (%) out of 594 for each communication topic.

^**ǂ**^ Chi-square test

SRH: Sexual and reproductive health.

No adjustment done for multiple testing.

**Fig 1:**
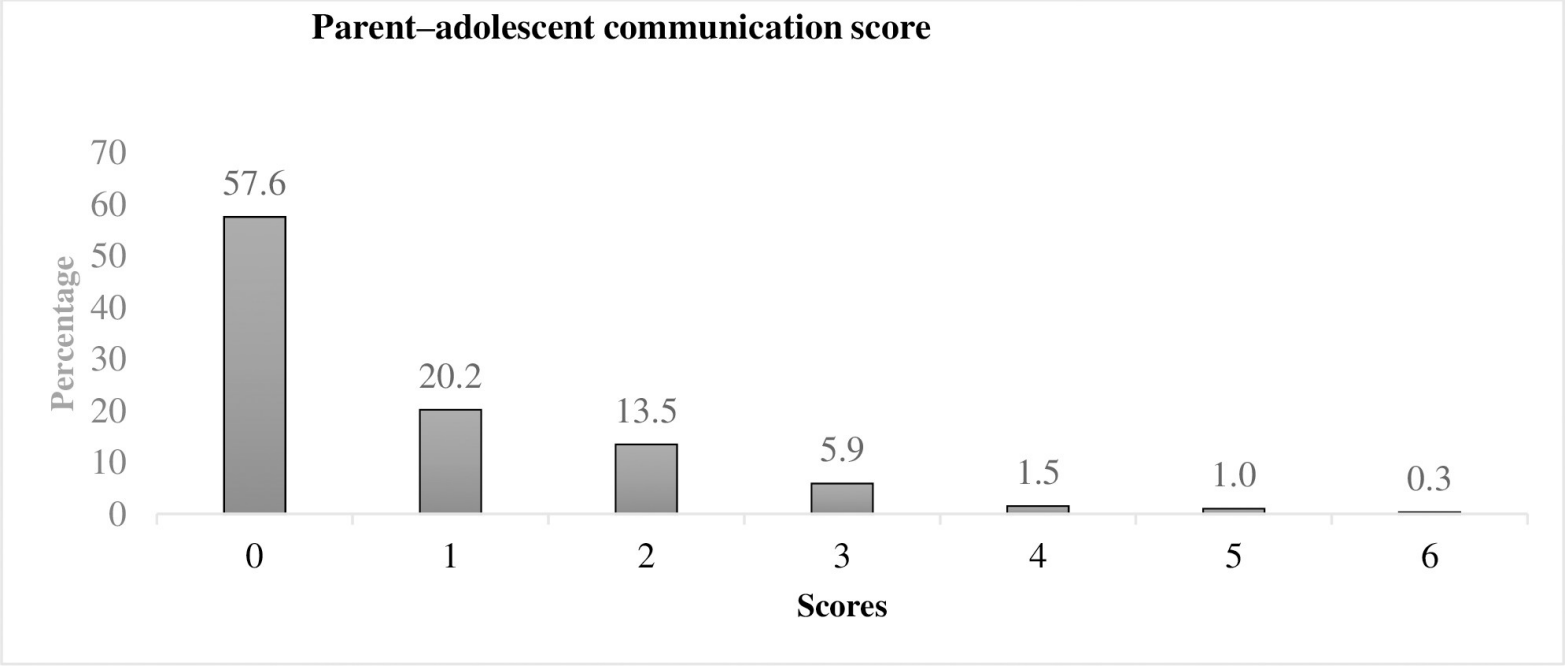
Parent–adolescent communication score (n = 594).

Fig 1 shows the distribution of parent–adolescent communication scores. The median score was 0 (interquartile range 1). About 57% of the students reported no communication with parents on SRH issues; whereas, 20.2%, 13.5%, and 5.9% of the students reported communication with parents on any one, two, or three of the seven SRH issues, respectively.

### Utilization of adolescent-friendly health services

Table 4 shows the utilization of adolescent-friendly health services by general characteristics (unadjusted analysis). Of the 594 students, 76 (12.8%) reported service utilization in the past one year. More male students used the services as compared to their female counterparts. The proportion of students who used services was greater among those from the Janajati ethnic group than those from the Brahmin/Chhetri and Dalit ethnic groups. More students living with both parents used services than those living alone or with others (siblings, other relatives, or friends). Service use was more common among students who reported having had sexual intercourse in the past year. There was no significant difference in service use based on parents’ education and wealth quintile.

**Table 4:** Utilization of adolescent-friendly health services (n = 594).

Variables	Utilization of adolescent-friendly health services				Test statistic	p-value	df
	Yes (n = 76)		No (n = 518)				
	N	%	N	%			
Age (Mean, SD)*	17.3 (1.0)		17.4 (1.1)		-0.75	0.223	1
Sex**					7.15		1
Male	43	17.1	209	82.9		**0.008**	
Female	33	9.6	309	90.4			
Grade**					1.07		1
11	44	14.1	267	85.9		0.301	
12	32	11.3	251	88.7			
Type of school**					3.14		1
Public	62	14.5	365	85.5		**0.044**	
Private	14	8.4	153	91.6			
**Marital status**^**ǂ**^							1
Married	3	33.3	6	66.7	0.10	0.063	
Unmarried	73	12.5	512	87.5			
Residence**					3.34		1
Rural	17	18.7	74	81.3		0.068	
Urban	59	11.7	444	88.3			
Religion**					0.91		1
Hindu	70	12.5	491	87.5		0.340	
Christian/Muslim/Buddhist	6	18.2	27	81.8			
Ethnicity**					17.11		1
Brahmin/Chhetri	23	7.4	287	92.6		**<0.001**	
Janajati	44	19.2	185	80.8			
Dalit	9	16.4	46	83.6			
**Father’s education** (n = 590)**					2.63		2
No education	6	9.0	61	91.0		0.269	
Grade 1 to 10	58	14.4	345	85.6			
Intermediate and above	12	10.0	108	90.0			
**Mother’s education** (n = 592)**					3.95		2
No education	13	8.5	140	91.5		0.139	
Grade 1 to 10	58	14.7	336	85.3			
Intermediate and above	5	11.1	40	88.9			
**Living arrangement**^**ǂ**^					0.01		3
With both parents	61	14.6	357	85.4		**0.008**	
With single parent (either father or mother only)	5	6.4	73	93.6			
Alone	2	3.4	57	96.6			
Others	8	20.5	31	79.5			
Wealth quintile**					1.45		4
Lowest	15	12.6	104	87.4		0.836	
Second lowest	12	10.0	108	90.0			
Middle	15	12.7	103	87.3			
Second highest	18	15.0	102	85.0			
Highest	16	13.7	101	86.3			
Had sexual intercourse in the past year**					194.18		1
Yes	57	53.8	49	46.2		**<0.001**	
No	19	3.9	469	96.1			

SD: Standard Deviation; df: Degree of freedom

* independent samples t-test

** Chi-square test

^ǂ^ Fisher’s exact test.

**Fig 2:**
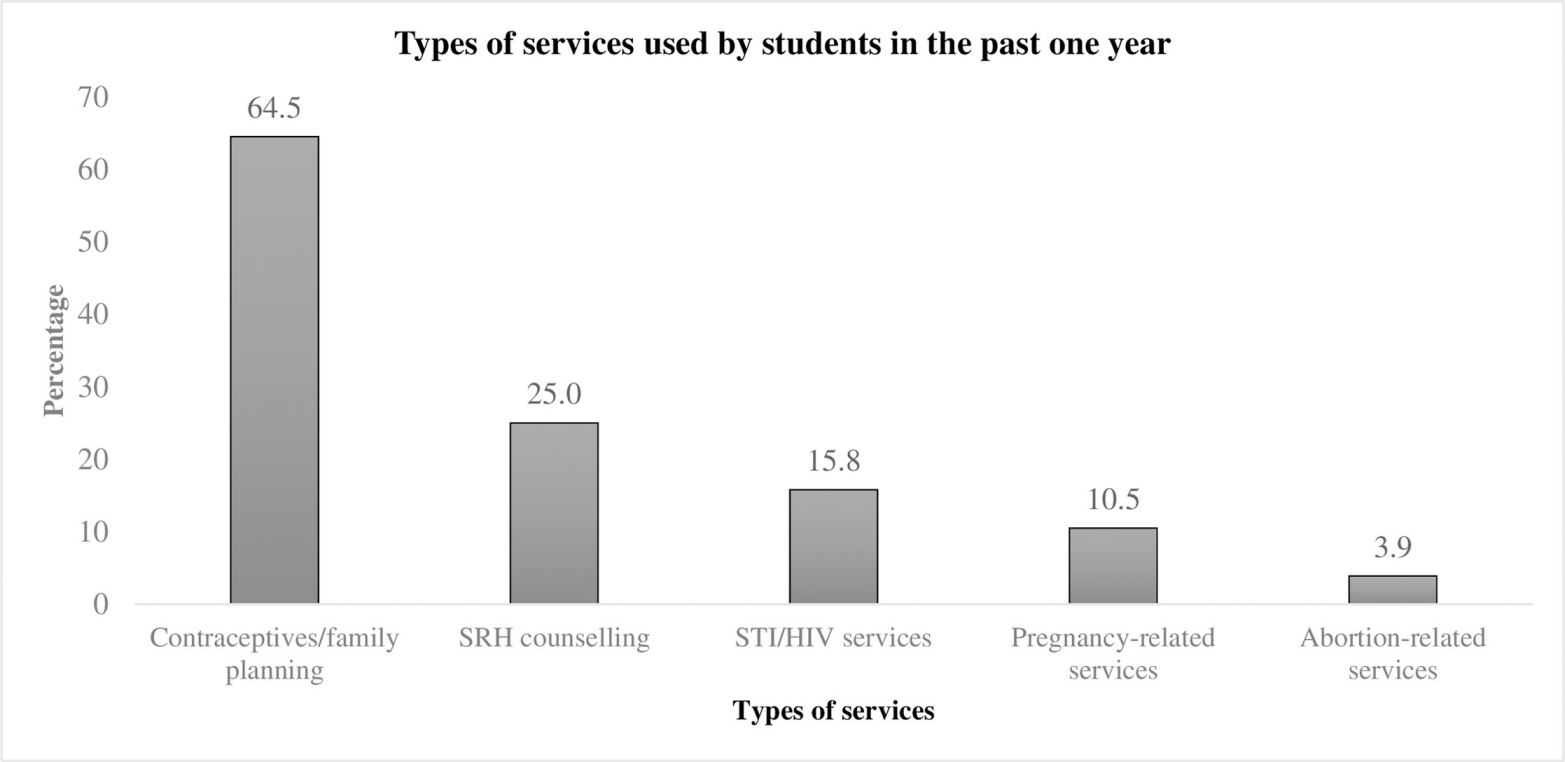
Types of services used by students in the past one year (multiple responses, n = 76).

Fig 2 displays the types of services used by students in the past one year. Contraceptives or family planning related services were used most commonly, followed by SRH information or counseling services, STI/HIV services, pregnancy-related services, and abortion-related services.

### Factors associated with the utilization of adolescent-friendly health services

The VIFs of all variables were below 10; therefore, no variable was excluded from the analyses. Table 5 shows the results of the multivariable logistic regression analysis on the utilization of adolescent-friendly health services and other variables (adjusted). Students with higher SRH communication scores were more likely to use adolescent-friendly health services (adjusted odds ratio, AOR: 1.70, 95% Confidence Interval, CI: 1.29–2.23, p<0.001).

**Table 5:** Factors associated with the utilization of adolescent-friendly health services (n = 588).

Variables	AOR	95% CI		p-value
**Parent-adolescent communication score**	1.70	1.29	2.23	**<0.001**
**Age**	0.90	0.64	1.28	0.570
**Sex**				
Male	1			
Female	0.56	0.25	1.29	0.175
**Residence**				
Rural	1			
Urban	0.89	0.25	3.11	0.854
**Marital status**				
Married	1			
Unmarried	1.50	0.24	9.54	0.665
**Grade**				
11	1			
12	0.83	0.42	1.65	0.597
**Type of school**				
Public	1			
Private	0.59	0.42	1.65	0.382
**Ethnicity**				
Brahmin/Chhetri	1			
Janajati	2.86	1.28	6.42	**0.011**
Dalit	2.53	0.75	8.56	0.136
**Religion**				
Hindu	1			
Christian/Muslim/Buddhist	1.37	0.35	5.31	0.650
**Father’s education**				
No education	1			
Grade 1 to 10	1.85	0.61	5.58	0.274
Grade 11 and above	0.79	0.13	4.60	0.789
**Mother’s education**				
No education	1			
Grade 1 to 10	1.81	0.72	4.60	0.210
Grade 11 and above	5.24	0.80	34.40	0.084
**Wealth quintile**				
Lowest	1			
Second lowest	1.02	0.30	3.43	0.979
Middle	1.46	0.36	5.86	0.596
Second highest	2.82	0.68	11.64	0.153
Highest	2.63	0.62	11.15	0.189
**Living arrangement**				
With both parents	1			
With single parent (either father or mother only)	0.42	0.12	1.50	0.182
Alone	0.12	0.02	0.66	**0.014**
Others	1.82	0.57	5.88	0.305
**Had sexual intercourse in the past year**				
No	1			
Yes	29.11	13.65	62.08	**<0.001**

AOR: Adjusted Odds Ratio; CI: Confidence Interval.

Adjusted for age, sex, residence, marital status, grade, type of school, ethnicity, religion, father’s education, mother’s education, wealth quintile, living arrangement, sexual behavior.

R-squared value: 0.45

Students from the Janajati ethnic group were more likely to use these services than those from the Brahmin/Chhetri ethnic group (AOR: 2.86, 95% CI: 1.28–6.42, p = 0.01). Moreover, those living alone were less likely to use services than those living with both parents (AOR: 0.12, 95% CI: 0.02–0.66, p = 0.01). Students who reported having had sexual intercourse in the past year were more likely to use services than those who did not report the same (AOR: 29.11, 95% CI: 13.65–62.08, p<0.001).

We obtained similar results even by using the multilevel logistic regression analysis with the random intercept at the classroom level. No variation was detected across classrooms.

## Discussion

Students with higher scores on SRH communication with parents were more likely to use adolescent-friendly health services in Kailali, Nepal. Furthermore, students living alone were less likely to use these services than those living with both parents. Belonging to the Janajati ethnic group and having had sexual intercourse in the past year were also positively associated with the utilization of adolescent-friendly health services.

In this study, a higher score on parent–adolescent communication about SRH was positively associated with increased SRH service utilization at health facilities. Students who have discussed such topics with their parents may want to maintain safer sexual behaviors [47, 48]. In Nepal, parents are known to play a significant role in improving adolescents’ intentions for safer sex [48]. Accordingly, adolescents who intend to have safer sex may utilize the adolescent-friendly health services offered. Furthermore, parent–adolescent communication about SRH may also have a positive effect on reducing risky sexual behaviors among adolescents [49]. Therefore, adolescents who communicated about such issues with their parents may want to utilize adolescent-friendly services, such as receiving condoms to prevent risky sexual behaviors. A study conducted in Ghana also supports the positive relationship between parent–adolescent communication and adolescents’ safer sexual behaviors. Specifically, that study found that adolescents who reported communication about HIV either with their parents or with any other family member were more likely to have used condoms during their most recent sexual intercourse [50].

Students with a higher score on parent–adolescent communication about SRH had greater odds of using adolescent-friendly health services in this study. Adolescents reporting sexuality communication with parents are also more likely to communicate about HIV with their sexual partners [51], and those who engage in such communication with their sexual partners are also more likely to use contraception services [52]. Thus, this study suggests that when adolescent students communicate with their parents and partners on SRH issues, they are more motivated to use preventive or treatment services on SRH.

Service utilization was less common among students living alone as compared to those living with both parents. In the present study, students living alone were mostly staying temporarily in the district, to seek better education. A study conducted in Ethiopia also showed that VCT service use was more common among adolescents living with both parents [23]. Another study found that female adolescents living with both parents had higher odds of using SRH services than those living with others [53]. Moreover, adolescents living alone may be less scared about the consequences of pregnancy or STIs as they have less opportunities to hear about them from their parents [54]. Thus, it may be more important to encourage service use among adolescents currently living alone or those not living with parents.

Service utilization was more common among students from the Janajati ethnic group than those from the Brahmin/Chhetri group. Sociocultural differences may influence adolescents’ general behavior in Nepal [55]. Culturally, the Brahmin/Chhetri ethnic group may have stricter norms related to SRH as compared to the Janajati ethnic group. Therefore, the difference in parent–adolescent communication or SRH service utilization could be attributed to cultural differences such as the degree of openness towards SRH issues. However, these findings may not be generalizable across the country, given the nature of variation within ethnic groups and differences in access to SRH services across different regions in Nepal. Further research is required to explain these differences in service utilization.

Finally, adolescent-friendly health service utilization was higher among students who reported having engaged in sexual intercourse in the past year. These adolescents could have higher SRH service needs than those who did not engage in sexual intercourse. Similar findings were reported among adolescents in Nigeria and Zambia [56, 57].

### Limitations and strengths

This study has the following limitations. First, due to the cross-sectional nature of this study, we cannot draw causal relationships, while reverse causality may be possible for the association between parent–adolescent communication and service utilization. For example, students who used the services might have communicated with their parents about SRH, depending on their needs. Second, since we did not have weighted data on sampling at the school level, we could not adjust for the probability of disproportional selection in the analysis. However, we also used a multilevel model that addressed the multi-stage nature of the sampling. Third, service utilization and sexual behaviors might have been underreported because of social desirability. To reduce this risk, we collected data in the classroom, in the absence of school authorities, and we ensured anonymity. Fourth, this study only included adolescents’ perspectives on SRH communication. Involving parents in the study may provide more insight on the details of SRH communication. Future studies may also consider investigating the timing and context of such communication. Fifth, this study does not represent non-school going adolescents whose parent–adolescent communication and service utilization may be different. However, the school was an appropriate site to collect data from majority of potential participants in this age group. Sixth, our study participants were mostly (98.5%) unmarried. Married students’ service utilization might be different from that of unmarried. Moreover, married female adolescent students are also more likely to dropout from school, and therefore, school-based studies may miss this population [58]. Consequently, there is still a chance that the present results do not reflect SRH service utilization in married adolescent students. Further, our participants were mostly from urban areas. Considering the urban–rural disparity in health services utilization, findings related to SRH utilization could also vary for rural settings. Finally, as this study was conducted only in one district, its findings may not be generalizable to the whole district or country. However, they could be applied to similar settings within the country and beyond.

Despite these limitations, the study has strengths. This study examined the underexplored association between parent–adolescent communication on SRH and the utilization of adolescent-friendly health services. The present findings could be used to design interventions for better utilization of adolescent-friendly health services.

## Conclusion

Students with higher parent–adolescent communication scores on SRH were more likely to use adolescent-friendly health services. Furthermore, those from the Janajati ethnic group and those who reported having engaged in sexual intercourse in the past year were more likely to use such services. In contrast, students who lived alone were less likely to use such services. Therefore, it may be desirable to involve parents in SRH communication with adolescents, as they could encourage the utilization of adolescent-friendly health services, and ultimately prevent negative SRH outcomes among students in late adolescence.

## Supporting information

S1 Table Multilevel logistic regression analysis for factors associated with the utilization of adolescent-friendly health services. (PDF)
