# Supplementary material for: Parent–adolescent communication on sexual and reproductive health and the utilization of adolescent-friendly health services in Kailali, Nepal
Source: PLoS One. 2021 Feb 19;16(2):e0246917. doi: 10.1371/journal.pone.0246917 (PMC7894935; doi:10.1371/journal.pone.0246917)
Supplement: S1 Table — (PDF) [file pone.0246917.s001.pdf]

**S1 Table. Multilevel logistic regression analysis for factors associated with the utilization of adolescent-friendly health services (n=588).**

| Variables | AOR | 95% CI | | p-value |
|---|---|---|---|---|
| **Parent-adolescent communication score** | 1.70 | 1.31 | 2.19 | **<0.001** |
| **Age** | 0.90 | 0.64 | 1.28 | 0.574 |
| **Sex** | | | | |
| Male | 1.00 | | | |
| Female | 0.56 | 0.25 | 1.24 | 0.152 |
| **Residence** | | | | |
| Rural | 1.00 | | | |
| Urban | 0.88 | 0.26 | 2.99 | 0.850 |
| **Marital status** | | | | |
| Married | 1.00 | | | |
| Unmarried | 1.50 | 0.22 | 9.98 | 0.673 |
| **Grade** | | | | |
| 11 | 1.00 | | | |
| 12 | 0.83 | 0.41 | 1.67 | 0.604 |
| **Type of school** | | | | |
| Public | 1.00 | | | |
| Private | 0.59 | 0.21 | 1.67 | 0.318 |
| **Ethnicity** | | | | |
| Brahmin/Chhetri | 1.00 | | | |
| Janajati | 2.86 | 1.20 | 6.85 | **0.018** |
| Dalit | 2.53 | 0.78 | 8.20 | 0.124 |
| **Religion** | | | | |
| Hindu | 1.00 | | | |
| Christian/Muslim/Buddhist | 1.37 | 0.37 | 5.13 | 0.642 |
| **Father's education** | | | | |
| No education | 1.00 | | | |
| Grade 1 to 10 | 1.85 | 0.51 | 6.73 | 0.350 |
| Grade 11 and above | 0.79 | 0.15 | 4.21 | 0.778 |
| **Mother's education** | | | | |
| No education | 1.00 | | | |
| Grade 1 to 10 | 1.18 | 0.74 | 4.46 | 0.195 |
| Grade 11 and above | 5.24 | 0.86 | 31.02 | 0.068 |
| **Wealth quintile** | | | | |
| Lowest | 1.00 | | | |
| Second lowest | 1.02 | 0.31 | 3.35 | 0.978 |
| Middle | 1.46 | 0.40 | 5.27 | 0.567 |
| Second highest | 2.82 | 0.79 | 10.01 | 0.110 |
| Highest | 2.63 | 0.67 | 10.35 | 0.166 |

| | | | | |
|---|---|---|---|---|
| **Living arrangement** | | | | |
| With both parents | 1.00 | | | |
| With single parent (either father or mother only) | 0.42 | 0.12 | 1.41 | 0.161 |
| Alone | 0.12 | 0.02 | 0.66 | **0.014** |
| Others | 1.84 | 0.55 | 6.11 | 0.321 |
| **Had sexual intercourse in the past year** | | | | |
| No | 1.00 | | | |
| Yes | 29.11 | 13.93 | 60.83 | **<0.001** |

| | | | | |
|---|---|---|---|---|
| **Random effect** | **Var** | **SE** | | |
| Classroom level | 1.18e-13 | 2.22e-07 | | |
| | **ICC** | **SE** | | |
| **Residual ICC** | 3.60e-14 | 0 | | |

AOR: Adjusted Odds Ratio; CI: Confidence Interval.
Adjusted for age, sex, residence, marital status, grade, type of school, ethnicity, religion, father's education, mother's education, wealth quintile, living arrangement, sexual behavior.
Var: Variance; ICC: Intraclass correlation.
